# Supplementary material for: Inheritance of OCT4 predetermines fate choice in human embryonic stem cells
Source: Mol Syst Biol. 2018 Sep 3;14(9):e8140. doi: 10.15252/msb.20178140 (PMC6120590; doi:10.15252/msb.20178140)
Supplement: Supplementary file 1 — Appendix [file MSB-14-e8140-s001.pdf]

Appendix for:

Inheritance of OCT4 predetermines fate choice in human embryonic stem cells

Samuel C. Wolff[1], Katarzyna M. Kedziora[1], Raluca Dumitru[1], Cierra D. Dungee[1], Tarek M. Zikry[2], Adriana S. Beltran[1], Rachel A. Haggerty[3], JrGang Cheng[4], Margaret A. Redick[1], and Jeremy E. Purvis[1,3,5,†]

[1]Department of Genetics
[2]Department of Biostatistics
[3]Curriculum for Bioinformatics and Computational Biology
[4]UNC Neuroscience Center
[5]Lineberger Comprehensive Cancer Center

University of North Carolina, Chapel Hill
120 Mason Farm Road
Chapel Hill, NC 27599-7264

[†]Corresponding Author:
Jeremy Purvis
Genetic Medicine Building 5061, CB#7264
120 Mason Farm Road
Chapel Hill, NC 27599-7264
jeremy_purvis@med.unc.edu

CONTENTS

Figure S1. hESCs with high OCT4 expression after BMP4 treatment are competent to differentiate into multiple cell types. 3

Figure S2. Expression of mesoderm-specific markers in response to BMP4 treatment 4

Experimental methods for constructing the H9 OCT4-mCherry cell line 5

Figure S3. OCT4-mCherry shows accurate co-localization, degradation kinetics, and chromatin binding patterns compared to endogenous OCT4 protein 7

Figure S4. Cells bearing the OCT4-mCherry reporter are competent to differentiate into multiple cell types 8

Figure S5. Proliferation of hESCs as a function of light exposure sampling rate and intensity 10

Figure S6. Classification of cell fates using a 2-component Gaussian distribution 11

Figure S7. Training results for logistic regression model 12

Table S1. Estimated coefficients and statistics for logistic regression model describing relationship between single-cell features and cellular fate. 13

Figure S8. Difference in OCT4 levels is strongly correlated with number of cell divisions separating two cells 14

Figure S9. A daughter cell’s inherited ratio of OCT4 becomes more strongly correlated with final OCT4 level over time 15

Figure S10. OCT4 ratios are not correlated with nuclear area or radial position within the colony 16

Figure S11. Autocorrelation analysis of OCT4 17

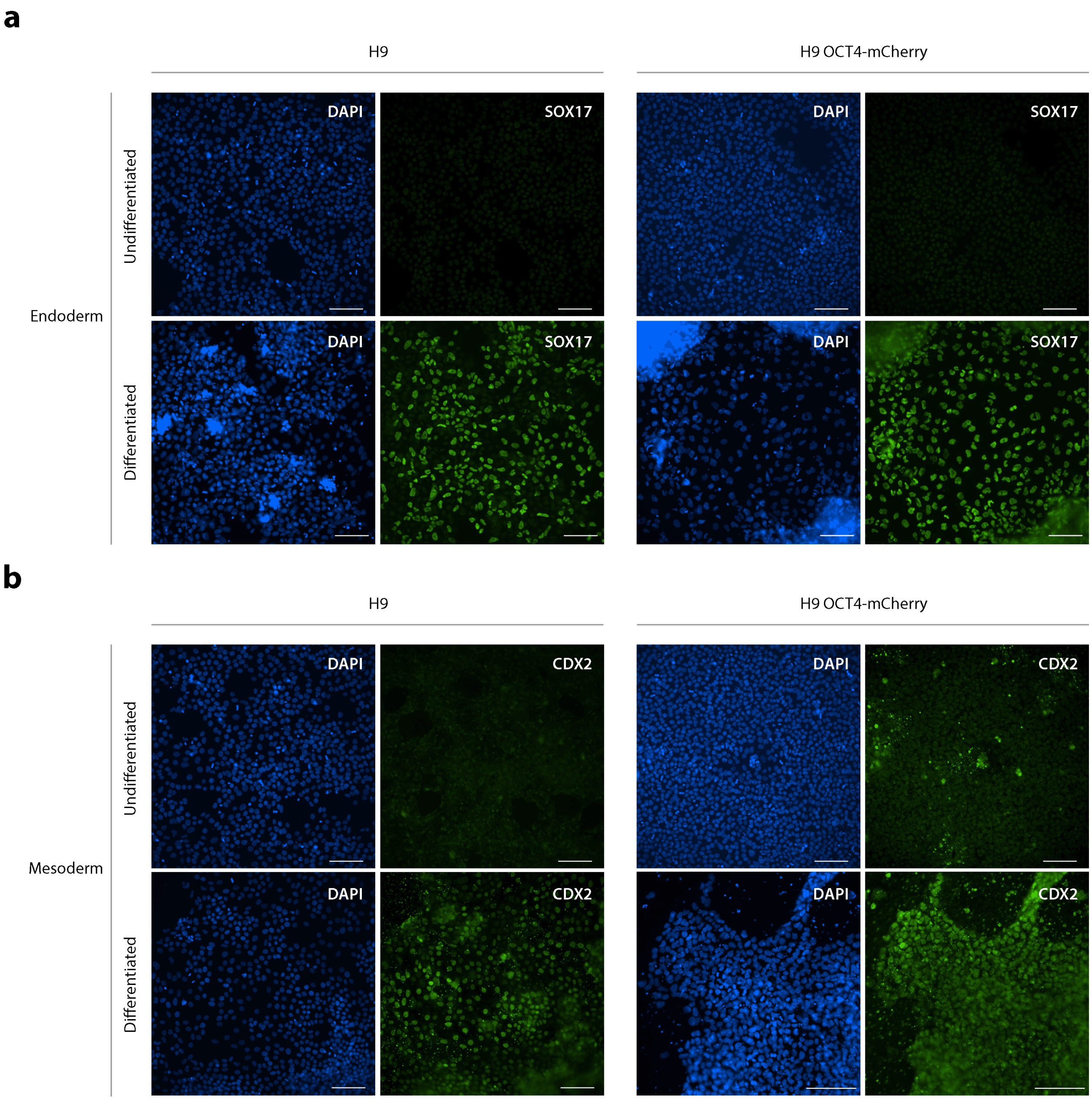

Figure S1. hESCs with high OCT4 expression after BMP4 treatment are competent to differentiate into multiple cell types. H9 wild-type and H9 OCT4-mCherry cells were seeded at a density of 2 × 10^5 cells/cm^2. After 1 d, cells were treated with 100 ng/ml BMP4 (Peprotech). After 24 hours, the center “core” of each colony (see Figure 2) was manually removed and placed on Matrigel-coated 24-well glass bottom plates. Cells were allowed to adhere for 2 hours and then differentiated toward **a,** endoderm or **b,** mesoderm using the STEMdiff™ Trilineage Differentiation Kit (STEMCELL Technologies). Scale bar = 100 μm.

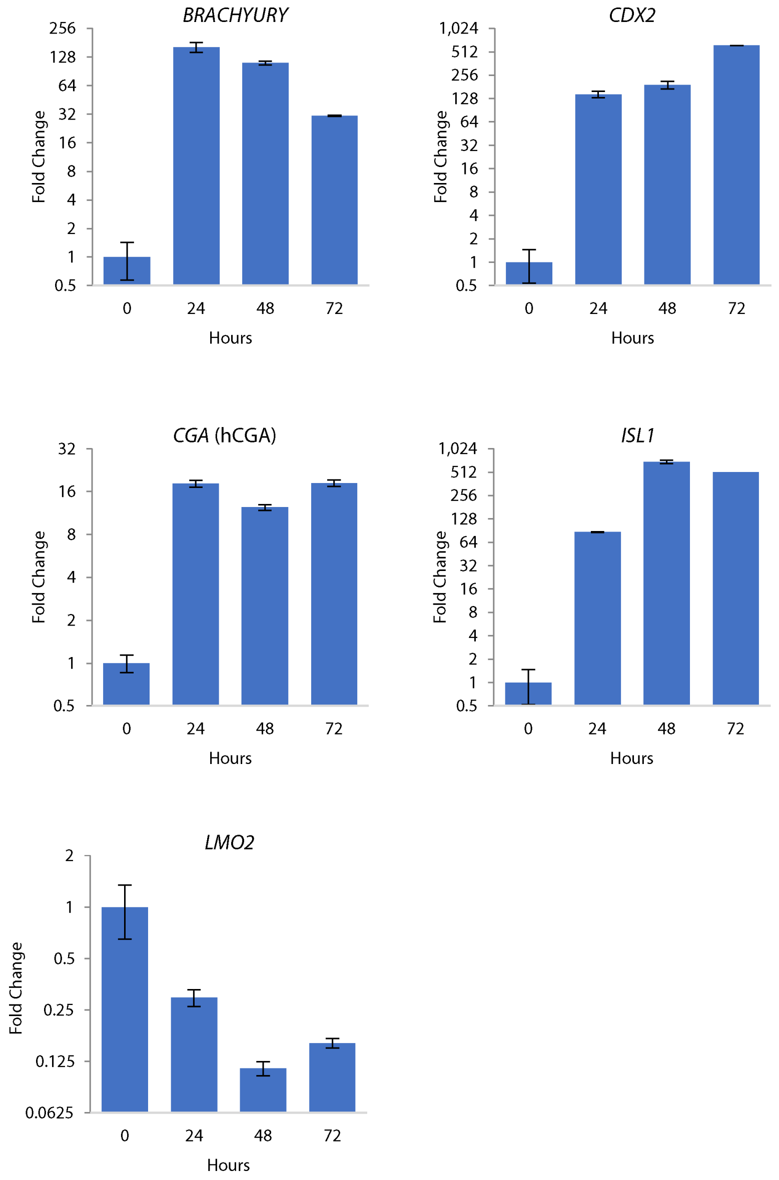

Figure S2. Expression of mesoderm-specific markers in response to BMP4 treatment. H9 hESCs were treated with 100 ng/mL BMP4 and harvested at 24, 48, and 72 h for quantification of transcript levels.

Experimental methods for constructing the H9 OCT4-mCherry cell line

Guide RNA and CRISPR/Cas9 cutting vector. The gRNA sequence GTGAAATGAGGGCTTGCGA, targeting the start codon of human *POU5F1* (OCT4), was cloned into pX330 (AddGene) using the standard cloning protocol described Ran et al(Ran et al, 2013). The cutting efficiency of the Cas9/OCT4-gRNA was validated with Guide-it Mutation Detection Kit (Takara Bio).

Donor cassette construction. The 5' homology arm of OCT4 was amplified out of H9 genomic DNA with the following primers (Fwd: 5'-AAGGTTGGGAAACTGAGGCC-3', Rev: 5'-GGGAAGGAAGGCGCCCCAAG-3) yielding a 1114 bp homology arm that was then cloned into the pGEMTEZ plasmid (Promega) followed by the coding sequence for the mCherry fluorescent protein (minus its stop codon) followed by a short linker sequence (TCC GGA TCC) and the start ATG codon for OCT4. The OCT4 gene constituted the 3' homology arm and was amplified out of H9 genomic DNA with the following primers (Fwd: 5'- ATGGCGGGACACCTGGCTTC-3', Rev: 5- AGCTTTCTACAAGGGGTGCC-3') yielding a 1082 bp homology arm.

Introduction of exogenous DNA into H9 cells. H9 cells were cultured on 10 cm dishes and, when 80% confluent, were dissociated using 0.5mM EDTA. 10×10^6 cells were resuspended in 800 µL ice-cold PBS containing 25 µg of the OCT4-mCherry donor vector and 25 µg of the guideRNA/Cas9 vector. Cells were electroporated in 100 µL tips (Neon, ThermoFisher Scientific) using program 19 of the optimization protocol (1050V, 30ms, 2 pulses) and resuspended in mTeSR1 (STEMCELL Technologies) supplemented with Rock inhibitor (S1049, Selleck Chemicals) at a final concentration of 10 µM. When colonies that expressed mCherry reached the size of a nickel, they were marked and picked into Matrigel coated 24-well plates.

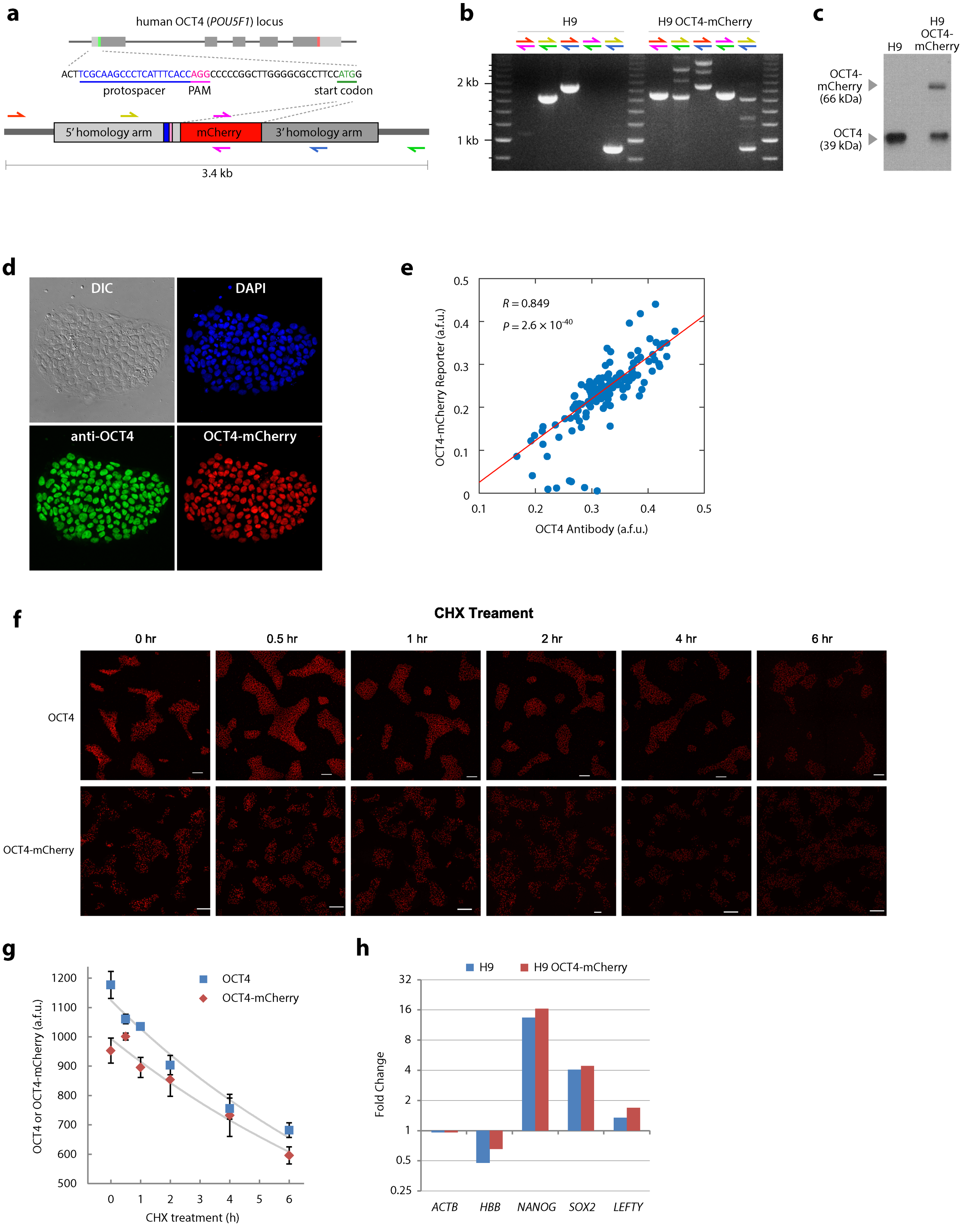
a
human OCT4 (POU5F1) locus
ACTTCGCAAGCCCTCATTTCACCAGGCCCCCGGCTTGGGGCGCCTTCCATGG
protospacer
PAM
start codon
5′ homology arm
mCherry
3′ homology arm
3.4 kb
b
H9
H9 OCT4-mCherry
2 kb
1 kb
c
H9
H9 OCT4-mCherry
OCT4-mCherry (66 kDa)
OCT4 (39 kDa)
d
DIC
DAPI
anti-OCT4
OCT4-mCherry
e
R = 0.849
P = 2.6 × 10^-40
OCT4-mCherry Reporter (a.f.u.)
OCT4 Antibody (a.f.u.)
0.5
0.4
0.3
0.2
0.1
0
0.1
0.2
0.3
0.4
0.5
f
CHX Treament
0 hr
0.5 hr
1 hr
2 hr
4 hr
6 hr
OCT4
OCT4-mCherry
g
OCT4
OCT4-mCherry
OCT4 or OCT4-mCherry (a.f.u.)
1200
1100
1000
900
800
700
600
500
0
1
2
3
4
5
6
CHX treatment (h)
h
H9
H9 OCT4-mCherry
Fold Change
32
16
8
4
2
1
0.5
0.25
ACTB
HBB
NANOG
SOX2
LEFTY

Figure S3. OCT4-mCherry shows accurate co-localization, degradation kinetics, and chromatin binding patterns compared to endogenous OCT4 protein.

a, A fluorescent mCherry coding sequence was introduced into the endogenous OCT4 locus of H9 hESCs using CRISPR-mediated homologous recombination. Primer pairs used for genomic validation are shown beneath the mCherry donor cassette. **b,** PCR Validation of genomic integration of OCT4-mCherry reporter in H9 cells using primer pairs shown in **a**. **c,** Western blot analysis of H9 and H9 OCT4-mCherry heterozygous cell line.**d,** Colony of clonal hESCs expressing OCT4-mCherry. **e,** Correlation between OCT4-mCherry reporter and endogenous OCT4 in single cells. Cells from Panel A were segmented and mean fluorescence intensity was quantified using CellProfiler (Carpenter et al, 2006). **f,** H9 or H9-OCT4-mCherry cells were seeded at a density of 3.75×10^4 cell/cm^2 in 12-well plates using mTeRS1 medium supplemented with 5 μM Y-27632 dihydrochloride ROCK inhibitor (STEMCELL Technologies). After 1 d, cells were treated with 50 μg/mL cyclohexamide for the indicated times and either imaged immediately (H9-OCT4-mCherry) or prepared for immunofluorescence imaging (H9). **g,** Mean fluorescence intensity was quantified for individual cells using Nikon Elements analysis software, and a mean-population intensity was calculated for each field of view by averaging the mean nuclear intensities over all cells in the field. Each data point is the average mean-population intensity of 3 fields of view, each containing approximately 1500 cells. Background-subtracted values were fit to an exponential function to yield half-life estimates of 8.65 ± 2.23 h for endogenous OCT4 and 7.34 ± 1.10 h for OCT4-mCherry. **h,** Enrichment of gene promoter regions for H9 hESCs and H9 OCT4-mCherry cell lines. Chromatin immunoprecipitation for OCT4 was performed as described by Jung et al(Jung et al, 2010). Primers for promoter regions were *ACTB*: ACTCGAGAGAG and CTACTACTCTAC; *HBB*: ACTCGAGAGAG and CTACTACTCTAC; *NANOG*: ACTCGAGAGAG and CTACTACTCTAC; *SOX2*: ACTCGAGAGAG and CTACTACTCTAC; and *LEFTY*: *ACTB*: ACTCGAGAGAG and CTACTACTCTAC.

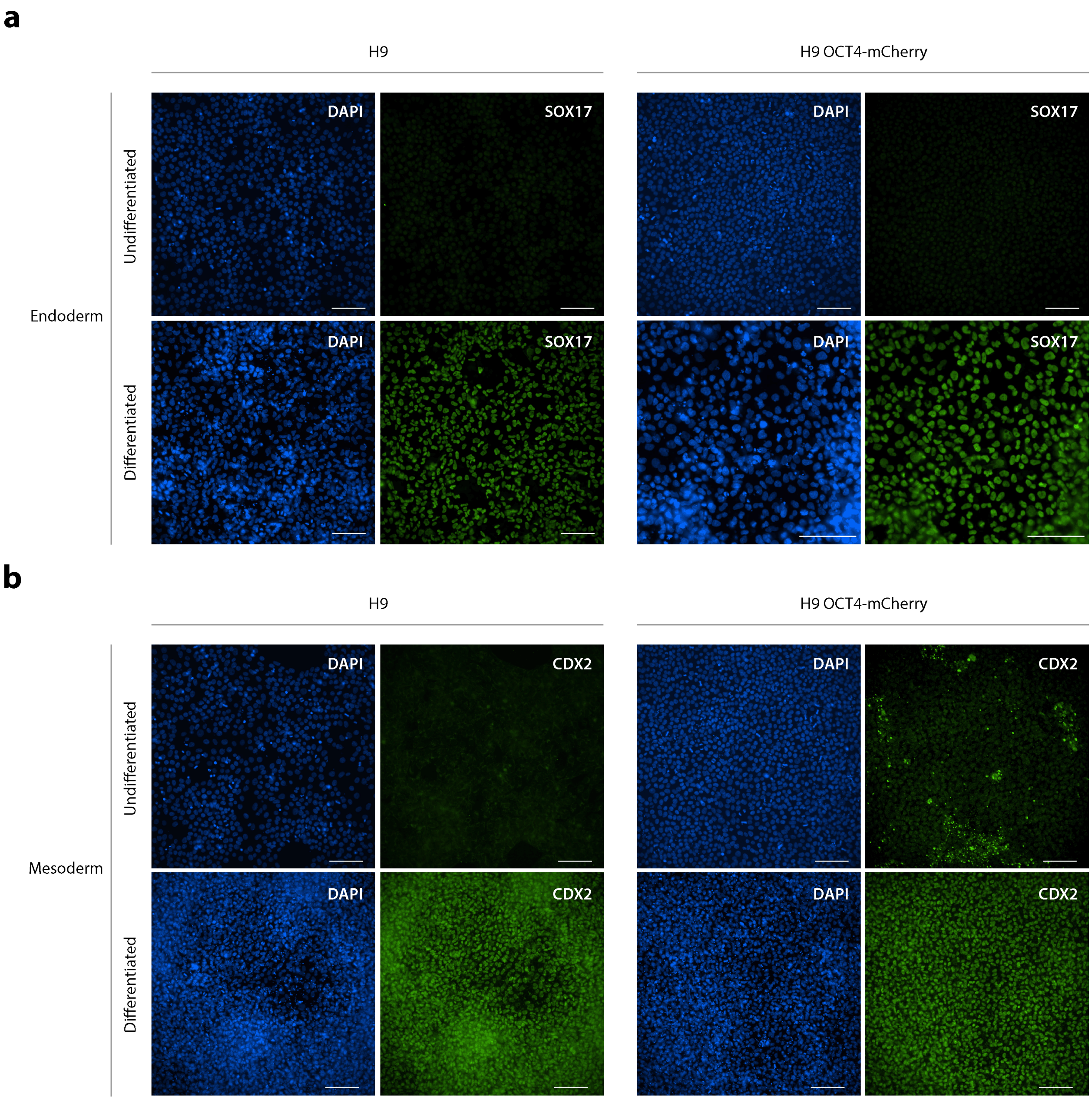

Figure S4. Cells bearing the OCT4-mCherry reporter are competent to differentiate into multiple cell types. H9 wild-type and H9 OCT4-mCherry clone 8-2 cells were seeded at the density of 2 × 10^5 cell/cm^2 for endoderm and 5 × 10^4 cell/cm^2 for mesoderm lineage differentiation on 24-well glass bottom plates using mTeRS1 medium supplemented with 5 µM Y27632 (STEMCELL Technologies). After 24 hours, cells were differentiated into **a,** endoderm or **b,** mesoderm using the STEMdiff™ Trilineage Differentiation kit (STEMCELL Technologies). Medium was replaced every day for 5 days, then cells were fixed 15 min with 4% paraformaldehyde, permeabilized for 15 min with 0.3% Triton X-100 and blocked 1 hour with 5% BSA at room temperature. Then, cells differentiated to endoderm were incubated with SOX17

(Abcam), and cells differentiated to mesoderm were incubated with CDX2 (Abcam), overnight. Cells were incubated with secondary antibody for 1 hour at room temperature. Nuclei were visualized with NucBlue Fixed Cell Stain ready Probes reagent (Molecular Probes). Scale bar = 100 µm.

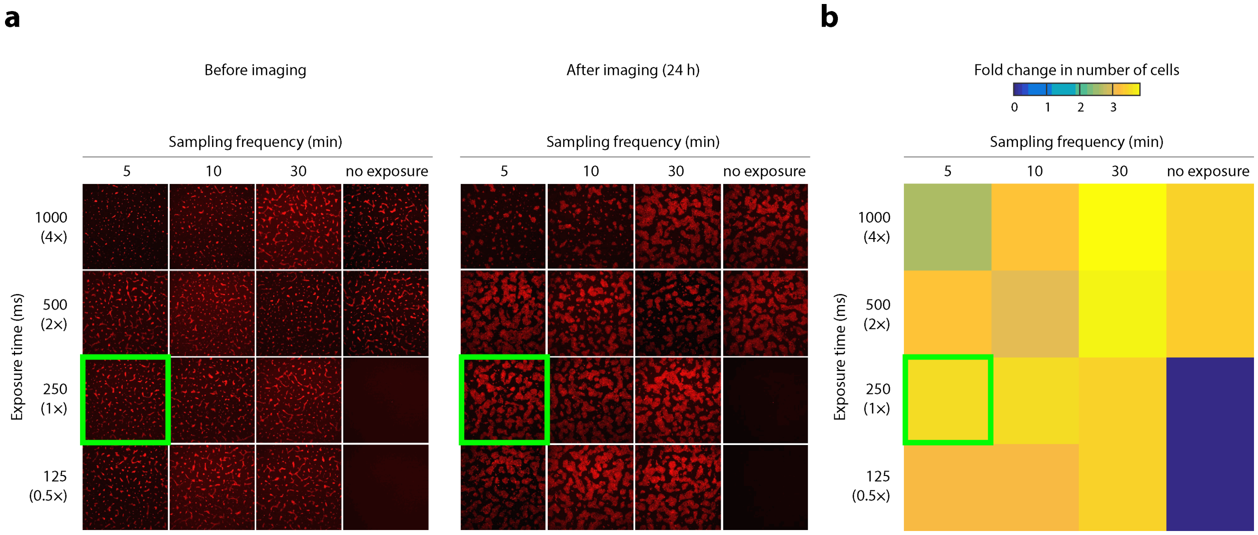

Figure S5. Proliferation of hESCs as a function of light exposure sampling rate and intensity. a, H9 OCT4-mCherry cells were seeded onto glass bottom dishes and subjected to time-lapse fluorescence imaging for 24 h at the indicated exposure durations and sampling frequencies. For each condition, a single image was captured by the camera both before and after light exposure to determine the extent of cell proliferation. Nikon Elements spot detection algorithm was used to count nuclei before and after imaging for each condition. The top two images in the fourth column are positive controls showing cells that were not exposed to any light other than during the first and last image captured for quantification of cell proliferation. The bottom two images in the fourth column are negative controls showing regions of the well that do not contain any cells. The green box indicates the exposure setting used in this study. **b,** Heat map showing the fold change in the number of nuclei quantified for each condition.

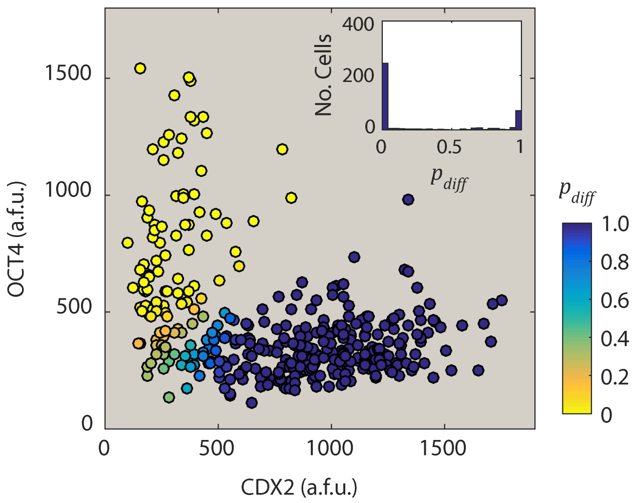

Figure S6. Classification of cell fates using a 2-component Gaussian distribution. Mean nuclear intensity values for OCT4 and CDX2 were used to separate the population of cells into two groups using a mixed Gaussian model. *Inset*, distribution of posterior probabilities (p_{diff}) for all cells. Only cells that could be confidently labeled as pluripotent (p_{diff}<0.01) or differentiated (p_{diff}> 0.99) were considered for pro-fate analysis. Similar class assignments were made by employing *k*-means clustering with *k* = 2.

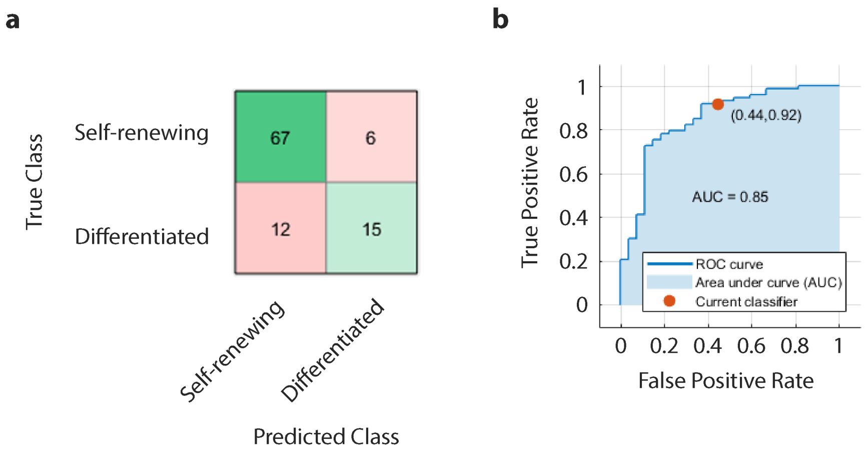

Figure S7. Training results for logistic regression model. a, Confusion matrix showing assignment of cells to self-renewing or differentiated classes. **b,** Receiver operating characteristic (ROC) plot for logistic regression classifier used to assign single cells to a pro-fate based on OCT4 dynamics and cell cycle duration. A comparison of all 7 possible models for 3 predictors revealed that OCT4 level was the strongest predictor of cell fate whereas burst frequency and cell cycle duration had poor predictive power on their own. Single-cell predictors and responses are described in the main text, the legend to **Figure 2**, **Table S1**, and the **Materials and Methods**.

Single-Cell Feature	Estimated Coefficient	Standard Error	t Statistic	P-Value
Levels	-0.0083047	0.0017799	-4.6657	3.0759e-06
Frequency	7.3216	2.7649	2.6481	0.0080949
Lifetime	0.043395	0.22411	0.19364	0.84646

Table S1. Estimated coefficients and statistics for logistic regression model describing relationship between single-cell features and cellular fate.

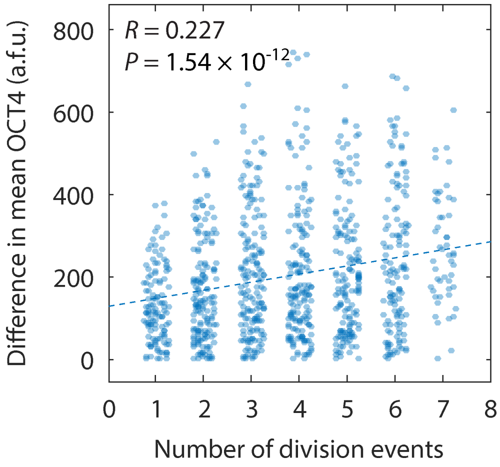

Figure S8. Difference in OCT4 levels is strongly correlated with number of cell divisions separating two cells. All pairs of cells in Figure 3a from the main text were compared for difference in OCT4 level (*y-axis*) and number of cell division events separating those cells (*x-axis*). For example, mother and daughter cell pairs are separated by 1 cell division event, whereas cousin cells are separated by 3 division events. As the number of division events increases, the difference in OCT4 levels off because each division event is equally likely to increase or decrease the difference in OCT4 levels between two given cells.

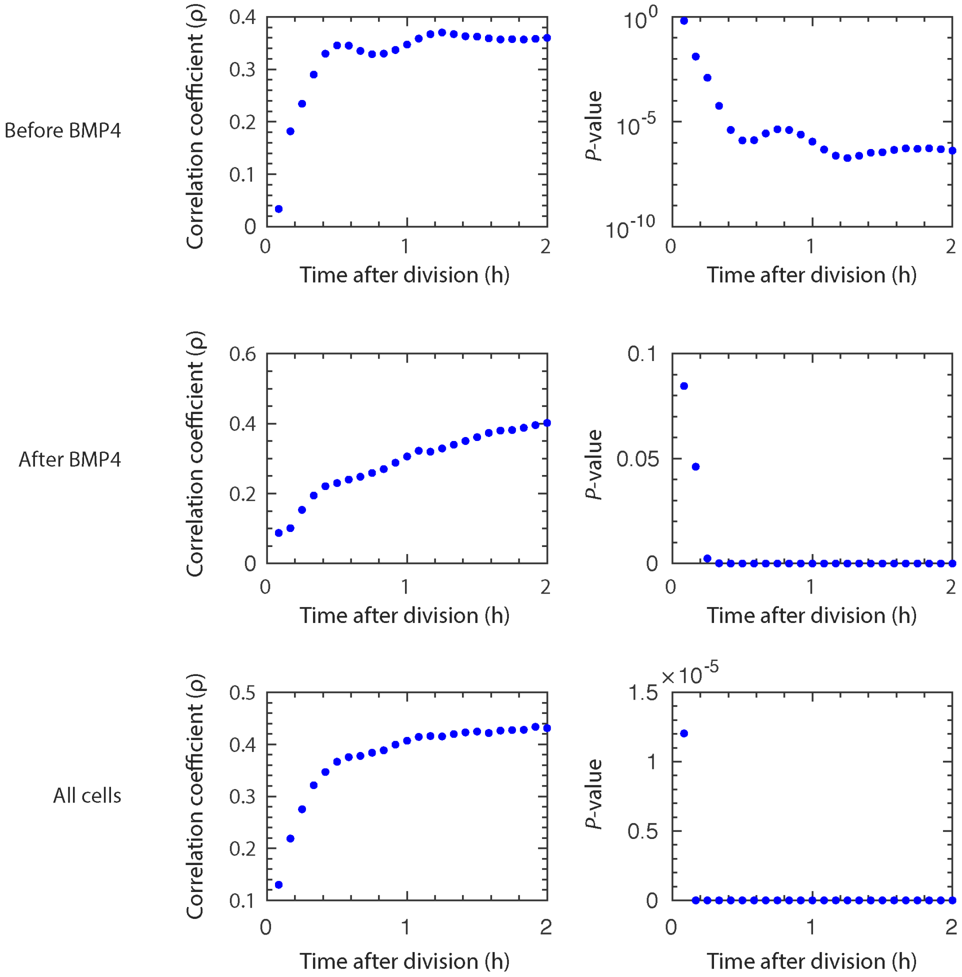

Figure S9. A daughter cell's inherited ratio of OCT4 becomes more strongly correlated with final OCT4 level over time. a, Correlation of OCT4 ratio between sister cells and differences in final OCT4 levels. At each time point, the ratio of OCT4 between sister cell pairs was calculated as the ratio of the cumulative sum of mean OCT4 intensity values at all preceding time points. This ratio was used to predict the final OCT4 level for each cell, calculated as the cumulative sum of mean intensities for the remaining time points. **b,** Corresponding *P*-values for correlation in panel **a**.

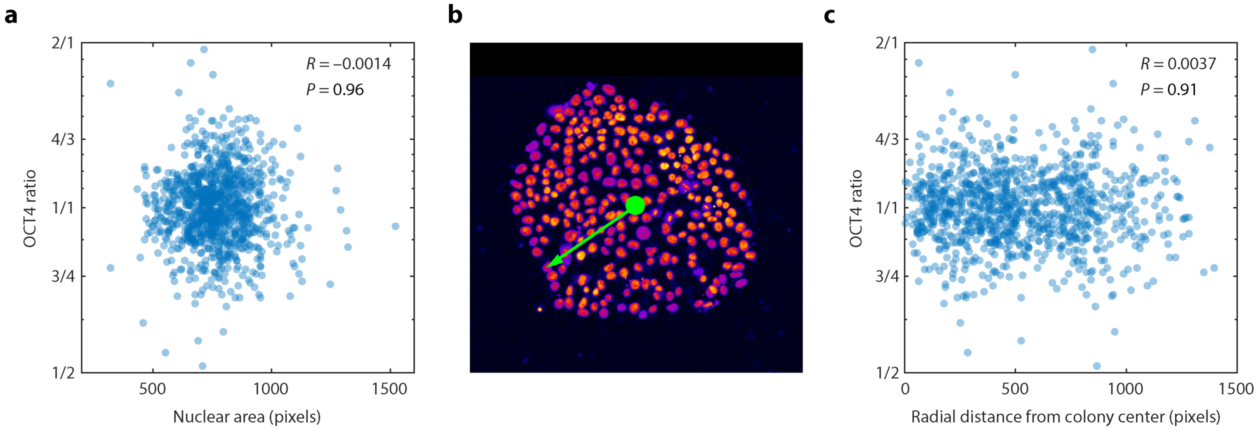

Figure S10. OCT4 ratios are not correlated with nuclear area or radial position within the colony. a, Scatter plot of OCT4 ratio as a function of nuclear area for sister cells. To avoid noise due to segmentation errors, nuclear area was calculated 25 min after cell division, when OCT4 became diffuse throughout the nucleus. **b,** The colony center was defined as a center of mass of all OCT4-expressing cells. The radial position of individual cells was defined as a distance (in pixels) between the center of a cell and the center of the colony. **c,** Scatter plot of OCT4 ratio as a function of radial distance from the center of the colony.

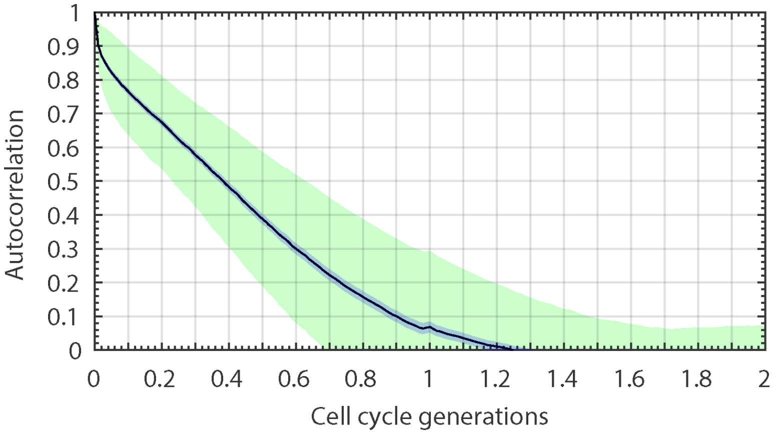

Figure S11. Autocorrelation analysis of OCT4. Total OCT4 expression traces were aligned to a common cell cycle timeline of 100 time points and concatenated over multiple cell cycle generations. Autocorrelation was calculated as described in (Sigal et al, 2006). Green shading denotes standard deviation and blue shading denotes standard error of the mean.

REFERENCES

Carpenter AE, Jones TR, Lamprecht MR, Clarke C, Kang IH, Friman O, Guertin DA, Chang JH, Lindquist RA, Moffat J, Golland P, Sabatini DM (2006) CellProfiler: image analysis software for identifying and quantifying cell phenotypes. *Genome Biol* **7:** R100

Jung M, Peterson H, Chavez L, Kahlem P, Lehrach H, Vilo J, Adjaye J (2010) A data integration approach to mapping OCT4 gene regulatory networks operative in embryonic stem cells and embryonal carcinoma cells. *PLoS One* **5:** e10709

Ran FA, Hsu PD, Wright J, Agarwala V, Scott DA, Zhang F (2013) Genome engineering using the CRISPR-Cas9 system. *Nature protocols* **8:** 2281-2308

Sigal A, Milo R, Cohen A, Geva-Zatorsky N, Klein Y, Liron Y, Rosenfeld N, Danon T, Perzov N, Alon U (2006) Variability and memory of protein levels in human cells. *Nature* **444:** 643-646
